# A real-world study of dupilumab in patients with atopic dermatitis including patients with malignancy and other medical comorbidities

**DOI:** 10.1016/j.jdin.2024.01.002

**Published:** 2024-01-17

**Authors:** Dea Metko, Maha Alkofide, Mohannad Abu-Hilal

**Affiliations:** a Michael G. DeGroote School of Medicine, Hamilton, Canada; b Division of Dermatology, McMaster University, Hamilton, Canada

**Keywords:** atopic dermatitis, comorbidities, dupilumab, malignancy, real-world experience

## Abstract

**Background:**

Dupilumab is a monoclonal antibody approved for the treatment of moderate-to-severe atopic dermatitis (MtS-AD). Various clinical trials have established the effectiveness and safety of dupilumab for the treatment MtS-AD; however, the real-world experiences of patients treated with dupilumab with malignancy and other comorbidities are lacking.

**Objective:**

To assess the real-life effectiveness and safety of dupilumab in the treatment of MtS-AD within Canadian adult patient population, including those with other significant comorbidities such as malignancy.

**Methods:**

In this retrospective study, records of adult patients diagnosed with MtS-AD, with a Physician Global Assessment (PGA) score of 3 or 4, and treated with dupilumab for 52 weeks were reviewed and collected.

**Results:**

A total of 155 adult patients with atopic dermatitis (AD) treated with dupilumab were included in the study. Asthma was the most common comorbidity. One hundred twenty-three (80%) patients received either phototherapy and/or at least 1 systemic agent (methotrexate and cyclosporine) before initiation of dupilumab. PGA score of 0 or 1 was achieved by 64% of patients at week 52. Adverse effects including injection site reactions, ocular surface disease, facial and neck redness, and arthropathy occurred in 6%, 10%, 8%, and 6% of patients, respectively. Three patients continued receiving dupilumab throughout pregnancy, all maintaining PGA score of 0 or 1 with no impact on pregnancy, delivery, or the newborn. Twelve patients with prior or active malignancy were included, with no reported negative impact on malignancy.

**Conclusion:**

Dupilumab is an effective and safe option for patients with AD in real life, including patients with malignancy and other medical comorbidities.

**Capsule Summary**

• This article enhances our current understanding of the safety and efficacy of dupilumab use in patients with atopic dermatitis, particularly those with comorbidities and malignancy.
• By showcasing the real-world effectiveness of dupilumab, this article encourages clinicians to consider dupilumab for treatment of patients with atopic dermatitis and comorbidities, including malignancy.

## Introduction

Atopic dermatitis (AD) is a complex chronic inflammatory skin disease characterized by pruritus, xerosis, persistent and/or recurrent eczematous papules and plaques, and lichenification. In 2017, dupilumab was the first treatment approved in Canada for moderate-to-severe atopic dermatitis (MtS-AD). Dupilumab is a human monoclonal antibody that targets the interleukin 4 receptor-alpha (IL-4Rα) subunit shared by both interleukin 4 (IL-4) and IL-13 which are key cytokines in the pathogenesis of AD. Multiple clinical trials have demonstrated the effectiveness and safety of dupilumab for the treatment of MtS-AD.^1^ However, clinical trials might not provide a complete representation of the patient population or reflect the drug’s actual clinical efficacy and safety in real-life patients because of the strict inclusion and exclusion criteria for patients’ participation. The purpose of this study is to bridge the gap that exists between outcomes seen in clinical trials and the actual real-world experience by conducting a comprehensive retrospective analysis of patients’ records with AD including patients with other significant comorbidities, and to describe the effectiveness of dupilumab in real-life practice. Ultimately, the findings of this study could help health care providers make better informed decisions and tailor treatment approaches to better suit the rare needs and characteristics of patients they encounter in their daily practice.

## Methods and materials

### Data collection

Patient records at dermatology clinics at Hamilton Health Sciences Centre and/or McMaster University at Hamilton, Ontario, were searched from December 1, 2017, to July 31, 2023. Adult patients with clinical diagnosis of MtS-AD, with Physician Global Assessment (PGA) scores of 3 or 4, and who received dupilumab for 52 weeks were included. All patient identifying data were fully anonymized to ensure confidentiality. Patient data were extracted from electronic medical records into a secure, password-protected, anonymized electronic database. Data collected and compiled into a spreadsheet included demographics, age onset of AD, duration of AD, family history of AD or any atopic diathesis, comorbidities including asthma, allergic rhinitis, chronic idiopathic urticaria, prurigo nodularis, alopecia areata, eosinophilic esophagitis, chronic rhinosinusitis with or without nasal polyps, depression, anxiety, HIV, malignancy, multiple sclerosis (MS), rheumatic disease, hepatitis C or B, bullous pemphigoid, inflammatory bowel disease, chronic renal failure (CRF), and history of kidney transplant. Additionally, previous treatments used with methotrexate (MTX), cyclosporine, and narrowband UV-B (NB–UV-B) were also collected. Eczema Area and Severity Index (EASI) when available, and PGA scores before and at week 52 of dupilumab were also collected. Side effects including injection sites reactions (ISRs), headaches, dupilumab-induced ocular surface disease (DIOSD), dupilumab facial redness (DFR), serious infections, herpes zoster, oral herpes, arthralgia and/or arthropathy, new onset alopecia areata, and new onset eosinophilia were also gathered. Data on 3 patients who continued dupilumab treatment throughout the pregnancy were also included in the analysis.

### Statistical analysis

Continuous variables were reported as median with range and mean ± SD. Categorical variables were summarized as frequencies and percentages of the study population and by subgroups, where appropriate.

## Results

Table I summarizes demographic and clinical data collected. A total of 155 adult patients with AD treated with dupilumab at the approved dose were included in the study. Mean age ± SD was 40 ± 17.85 years (range, 18-86 years). Sixty-nine (45%) were male and 86 (55%) were female. For those with infantile or childhood onset, the mean age of AD diagnosis was 4.5 years, and the mean age for AD diagnosis for adult onset was 43 years of age. The average duration of AD for the whole cohort was 15.28 years. Twenty-nine (19%) reported family history of AD and 33 (21.3%) reported family history of at least 1 of the atopic diatheses (AD, asthma, or allergic rhinitis). Medical and mental health comorbidities are also summarized in Table I.

**Table I:** Demographic and clinical data for patient cohort

Patient data	Value
Female, *n* (%)	86 (55%)
Age (y), mean ± SD	40.2 ± 17.85
Mean age of onset of for all patients (y)	24.86
Mean age of onset for patients with infantile/childhood onset AD (y)	4.5
Mean age of onset for patients with adult onset AD (y)	43
Mean duration of disease (y)	15.28 ± 14.337
Family history AD, *n* (%)	29 (19%)
Family history of at least 1 of the atopic diathesis (asthma, allergic rhinitis, or AD), *n* (%)	33 (21%)
Asthma, *n* (%)	38 (25%)
Allergic rhinitis, *n* (%)	28 (18%)
CIU, *n* (%)	12 (8%)
Alopecia areata, *n* (%)	9 (6%)
Chronic rhinosinusitis with or without nasal polyps, *n* (%)	8 (5%)
Eosinophilic esophagitis, *n* (%)	2 (1%)
Depression, *n* (%)	34 (22%)
Anxiety, *n* (%)	20 (13%)
HIV, *n* (%)	1 (0.65%)
Malignancy, *n* (%)	12 (8%)
Multiple sclerosis, *n* (%)	2 (1%)
Rheumatic disease, *n* (%)	16 (10%)
Prurigo nodularis, *n* (%)	12 (8%)
Hepatitis C, *n* (%)	1 (0.65%)
Bullous pemphigoid, *n* (%)	1 (0.65%)
IBD, *n* (%)	2 (1%)
CRF, *n* (%)	1 (0.65%)
Kidney transplant recipient, *n* (%)	3 (2%)
Pregnancy, *n* (%)	3 (2%)
Previous treatments, *n* (%)	
MTX	94 (61%)
Cyclosporine	53 (34%)
NB–UV-B	39 (25%)

*AD*, Atopic dermatitis; *CIU*, chronic idiopathic urticaria; *CRF*, chronic renal failure; *IBD*, inflammatory bowel disease; *MTX*, methotrexate; *NB–UV-B*, narrowband UV-B.

One hundred twenty-three (79%) patients received either NB–UV-B and/or at least 1 systemic agent (MTX and cyclosporine) before initiation of dupilumab. MTX was the most commonly used agent before the usage of dupilumab by 94 (61%) of patients. Cyclosporine and NB–UV-B were used by 34% and 25% of patients, respectively, before dupilumab.

Ninety-nine (64%) patients achieved PGA score of 0 or 1 at week 52. EASI scores before and after treatment with dupilumab were available for 88 patients. Sixty-two (70%) of these 88 patients achieved EASI 75 at week 52. The average EASI score prior and after dupilumab were 22 and 4.3, respectively. Table II summarizes clinical and safety outcomes at week 52.

**Table II:** Clinical and safety outcomes at week 52

	Value, n (%)
Outcome	
Patients achieving PGA score of 0 or 1	99 (64%)
Patients achieving EASI 75 (when EASI score is available)	62/88 (70%)
Adverse effects	
Injection site reaction	9 (6%)
DIOSD	16 (10%)
DFR	12 (8%)
Arthralgia and/or arthropathy	9 (6%)
Alopecia areata	2 (1%)
Transient eosinophilia	13 (8%)
Oral herpes	1 (0.65%)
Herpes zoster	0

*DIOSD*, Dupilumab-induced ocular surface disease; *DFR*, dupilumab facial redness; *EASI*, Eczema Area and Severity Index; *PGA*, Physician Global Assessment.

Only 9 patients (6%) reported ISRs but no anaphylaxis or serious reactions were reported. All ISRs happened with the first injection only and never recurred. Sixteen (10%) patients experienced DIOSD. Median onset was 4 months from starting dupilumab. Only one patient discontinued dupilumab because of severe uncontrolled DIOSD including evidence of peripheral and central corneal infiltrates upon ophthalmic examination. DFR developed in 12 patients (8%), with median onset of 8 weeks (range, 2-42 weeks) after starting dupilumab. Three (2%) patients discontinued dupilumab because of DFR. Only one patient (0.65%) had oral herpes simplex but no cases of herpes zoster reported. Complete blood count was available for 30 patients during the course of dupilumab treatment. Mildly elevated eosinophil counts developed in 13 patients, with median onset of 8 weeks (range, 3-20 weeks) after starting dupilumab. However, all cases resolved with continuation of treatment.

Nine patients (6%) reported arthralgia during their treatment course. Interestingly, none of these patients had previously confirmed inflammatory arthritis. Median onset was 16 weeks (range, 12-24 weeks) after starting dupilumab. Three patients with persistent and severe arthralgia were evaluated by rheumatologists. One was eventually diagnosed with rheumatoid arthritis, and one was diagnosed with mild ankylosing spondylitis.

A total of 3 patients continued dupilumab throughout pregnancy, all patients maintained PGA score of 0 or 1. There was no reported impact on pregnancy, delivery, or the newborn to the time of writing this article.
Twelve patients with prior or active malignancy were treated with dupilumab. Table III summarizes patients with malignancy. None of the patients reported negative impact of malignancy to the time of writing the article.

**Table III:** Outcomes for patients with malignancy treated with dupilumab

Patient	Age (y)	Sex	Malignancy	Year of onset	Status of malignancy before starting dupilumab (in remission or actively being treated)	Negative impact reported
1	56	F	Breast	2010	Nonactive, in remission for 10 y	None
2	30	M	Testicular	2000	Nonactive, in remission for 20 y	None
3	19	F	Papillary thyroid carcinoma	2020	Nonactive, in remission for 2 y	None
4	35	F	Breast	2017	Nonactive, in remission for 3 y	None
5	28	F	NHL	2014	Nonactive, in remission for 6 y	None
6	26	M	AML	2006	Nonactive, in remission for 14 y	None
7	57	M	RCC	2015	Nonactive, in remission for 6 y	None
8	51	F	Breast	2012	Nonactive, in remission for 8 y	None
9	65	F	Ovarian	2011	Nonactive, in remission for 10 y	None
10	39	M	Melanoma	2022	Active and being treated	None
11	59	F	NHL	2006	Nonactive, in remission for 14 y	None
12	85	M	Prostate and RCC	2014 for prostate cancer and 2007 for RCC	Nonactive, in remission for 7 and 13 y, respectively	None

*AML*, Acute myeloid leukemia; *F*, female; *M*, male; *NHL*, non-Hodgkin lymphoma; *RCC*, renal cell carcinoma.

## Discussion

Here we present a large retrospective analysis of real-world adult patients with AD who received treatment with dupilumab in Canada. Several clinical trials have consistently demonstrated the efficacy of dupilumab in patients with MtS-AD. The LIBERTY AD CHRONOS specifically evaluated the long-term efficacy and safety of dupilumab in combination with medium-potency topical corticosteroids in adult patients with MtS-AD.^2^ In this trial, 39% and 36% of patients achieved a PGA score of 0 or 1 at weeks 16 and 52, respectively, our study showed that 63.9% of patients achieved a PGA score of 0 or 1 at week 52. Our results confirm that dupilumab is an effective and safe treatment option for AD in real-world settings with significant and sustained improvements in disease severity. The higher percentage of patients achieving PGA score of 0 or 1 in our study compared with LIBERTY AD CHRONOS trial is likely attributed to more liberal use, and higher potency of topical corticosteroids used in our patients, as well as the use of oral antibiotics and/or oral prednisone in combination with dupilumab in some patients, which was not captured in the data. Additionally, 4 patients received both MTX and dupilumab concurrently, and 2 patients used cyclosporine and dupilumab simultaneously for a period of 2 to 4 weeks before discontinuing the oral agents. Furthermore, 4 patients received more frequent dosing regimen of dupilumab, and 4 patients continued to receive NB–UV-B with dupilumab for 6 to 10 weeks before discontinuing phototherapy.

Previous literature has generally shown that dupilumab usage for AD is safe with no major adverse effects (AEs).^3^ AEs reported in previous clinical trials and real-world experience studies include ISRs, DIOSD, DFR, and arthropathy.^4^ ISRs are common AEs with an incidence rate of approximately 12.3% in groups receiving dupilumab 300 mg every 2 weeks in clinical trials.^5^ The low incidence of ISRs in our study (6%) compared with other studies may be attributed to factors such as underreporting by patients, particularly if the reactions are mild and do not require medical attention. Furthermore, in a clinical trial setting, strict and close follow-up and assessment of injection sites could also contribute to the early identification and management of ISRs, potentially leading to a lower reported frequency in our study.^6^

Our findings showed that DIOSD developed in 10% of patients, with only 1 patient discontinuing dupilumab. Comparatively, in previous literature, conjunctivitis and/or DIOSD, occurred at varying rates with some studies showing 4% to 12.5%,^6^, ^7^, ^8^, ^9^, ^10^, ^11^ whereas other studies reported significantly larger proportions (20%-34%) of their cohorts developing conjunctivitis.^12^, ^13^, ^14^, ^15^, ^16^, ^17^ It is worth mentioning that patients in our cohort were frequently informed about this potential AE, and were consistently advised to use lubricating eye drops as a prophylactic measure before and during the course of dupilumab therapy. This may explain the slightly lower rate of DIOSD in our cohort.

DFR is also a known side effect of dupilumab usage with a pathogenesis that is not fully understood. In our study 12 (7.7%) of patients encountered DFR. Previous real-world experience studies have reported a wide range of occurrence of DFR; between 4% and 43.8%.^6^^,^^7^ Although the incidence in our cohort falls within this reported range, we believe it might be underestimated. First, patients commonly resort to self-administration of topical corticosteroids or topical calcineurin inhibitors for management. Additionally, it is possible that DFR was not captured because of misinterpretation of DFR as a symptom of AD, or resistance to treatment, thus leading to its slightly underreporting in our findings. Waldman et al^18^ suggested that DFR does not warrant the cessation of treatment; however, 3 patients in our cohort discontinued dupilumab because of this AE aligning with similar observations reported in previous studies.^19^

Arthropathy is uncommon or potentially underrecognized potential AE for dupilumab. Retrospective studies have reported that some form of arthropathy is developed in 3.6% to 5.8% of patients after initiating dupilumab for patients with AD.^20^ The arthropathy may include arthralgia, inflammatory arthritis, enthesitis, and tenosynovitis.^20^^,^^21^ In our study, arthropathy was reported by 9 (6%) of patients. The slightly high number is likely because of the increased awareness of the treating dermatologists of this potential AE and regular inquiries about joint symptoms during each visit. Similar to existing literature, all our patients reported arthropathy within 6 months of starting dupilumab with median onset of 4 months.^22^ Apart from 3 patients, all reported mild and transient symptoms resolving with over-the-counter analgesics.

The safety of dupilumab warrants special consideration in specific populations particularly, in pregnant patients, and in patients with previous or current malignancies. Since AD may be exacerbated during pregnancy, evaluating the safety profile of dupilumab in this context is very crucial. In our study, all 3 patients who continued dupilumab throughout pregnancy achieved and maintained PGA score of 0 or 1. Furthermore, no AEs on pregnancy, delivery, or the newborn were reported. At present, there are limited data available on the effects of dupilumab on pregnancy and breastfeeding. Multiple case reports have reported positive treatment outcomes with dupilumab administration during pregnancy.^23^^,^^24^ Recently, Escolà et al^25^ described 11 pregnant women giving birth to 12 babies. One gave birth to 2 premature babies with low birth weight. However, this outcome is common in twin deliveries. Otherwise, no AEs were reported on either the women or their babies. Additionally, animal studies have shown no AEs and no evidence of fetal abnormalities associated with dupilumab.^26^ Furthermore, the rate of spontaneous abortion among patients treated with dupilumab appears comparable to that observed in the general population.^26^ Overall, there is still a pressing need for additional studies to further investigate the effects of dupilumab on pregnancy and newborns.

In our study, a total of 12 patients with previous malignancies, 11 patients in remission and 1 patient currently receiving active treatment for malignancy, received dupilumab treatment for AD. No adverse impact on malignancy was reported at the time of writing this article but long-term follow-up is needed for comprehensive safety evaluation. Our findings are consistent with existing literature with no recurrence of prior malignancy reported with the use of dupilumab.^27^ Interestingly, there is growing evidence that patients with active malignancy can safely benefit from dupilumab for AD.^28^ Additionally, a study evaluating the use of dupilumab for immune-related cutaneous adverse events in patients with malignancy reported no significant short-term safety concerns. Although our study included patients with acute myeloid leukemia, non-Hodgkin lymphoma, breast, kidney, thyroid, and testicular cancers, evidence of success with dupilumab usage has been observed in various other cancer types such as colorectal, prostate, melanoma, and squamous cell carcinoma.^27^^,^^29^ Furthermore, the International Eczema Council recommends the use of dupilumab as a first-line therapy for patients with AD and history of malignancy.^30^^,^^31^ Despite being excluded from clinical trials, our study demonstrates that this patient population can benefit from dupilumab use with minimal safety concerns.^32^

The safety profile of dupilumab extends further to special populations with rarer diseases such as MS, viral hepatitis, HIV, and CRF. In our study, there were no reported complications related to dupilumab usage in the 2 patients with MS. Although there is limited literature regarding usage of dupilumab for patients with AD and MS, 1 case series found that there were no changes in MS activity or magnetic resonance imaging results during the treatment’s use.^33^ However, 2 case studies reported patients developing MS after starting dupilumab for AD.^34^ Although dupilumab is generally considered safe for use in patients with AD and MS, the limited literature warrants continued caution and close liaison with neurologists in this specific population.^33^

There are also very limited data on the safety of dupilumab usage in patients with hepatitis, HIV, and CRF. However, existing case studies for patients with hepatitis B, hepatitis C, CRF, and HIV have found dupilumab to be safe and effective in these special populations.^32^^,^^34^, ^35^, ^36^, ^37^ However, more research is needed to better understand the safety profile of dupilumab in these special patient populations.^32^

Overall, our study has shown that dupilumab is an effective and safe treatment option for AD in real-life settings including patients with malignancy and other special populations. The clinical outcomes were comparable to those observed in clinical trials. However, it is essential to acknowledge the limitations of this study including the retrospective design, the relatively small population size, and lack of quality control surrounding data collection in real-world settings. Larger cohorts, longer follow-up, and more diverse patient populations are needed for better understanding of dupilumab for patients with AD in real-life practice.

## Conclusion

The findings of our study align with results from previous clinical trials and other real-world studies by showing that dupilumab is an effective and safe option for patients with AD. As more studies continue to monitor the efficacy of dupilumab in patients with AD and additional comorbidities, clinicians can make more informed and evidence-based decisions to tailor treatment and improve treatment outcomes.

## Conflicts of interest

Dr Alkofide has served as an adviser/consultant for, or received grants/honoraria from, or has served as a speaker for AbbVie, Eli Lilly, Galderma, Janssen, LEO Pharma, L’Oreal, Medexus, Novartis, Pfizer, Sanofi, and Sun Pharma. The other authors have no conflicts of interest to declare.
